# Life-long brain compensatory responses to galactic cosmic radiation exposure

**DOI:** 10.1038/s41598-021-83447-y

**Published:** 2021-02-22

**Authors:** Omid Miry, Xiao-lei Zhang, Linnea R. Vose, Katisha R. Gopaul, Galadu Subah, Juliet A. Moncaster, Mark W. Wojnarowicz, Andrew M. Fisher, Chad A. Tagge, Lee E. Goldstein, Patric K. Stanton

**Affiliations:** 1 Department of Cell Biology & Anatomy, New York Medical College, Valhalla, NY USA; 2 Department of Psychiatry, Boston University School of Medicine, Boston, MA USA; 3 Department of Pathology and Laboratory Medicine, Boston University School of Medicine, Boston, MA USA; 4 Department of Biomedical Engineering, Boston University College of Engineering, Boston, MA USA; 5 Molecular Aging & Development Laboratory, Boston University School of Medicine, Boston, MA USA; 6 Boston University Alzheimer’s Disease Center, Boston, MA USA; 7 Department of Cell Biology & Anatomy, Basic Sciences Building, New York Medical College, Room 217, 15 Dana Road, Valhalla, NY 10595 USA

**Keywords:** Neuroscience, Regeneration and repair in the nervous system, Synaptic plasticity

## Abstract

Galactic cosmic radiation (GCR) composed of high-energy, heavy particles (HZE) poses potentially serious hazards to long-duration crewed missions in deep space beyond earth’s magnetosphere, including planned missions to Mars. Chronic effects of GCR exposure on brain structure and cognitive function are poorly understood, thereby limiting risk reduction and mitigation strategies to protect against sequelae from exposure during and after deep-space travel. Given the selective vulnerability of the hippocampus to neurotoxic insult and the importance of this brain region to learning and memory, we hypothesized that GCR-relevant HZE exposure may induce long-term alterations in adult hippocampal neurogenesis, synaptic plasticity, and hippocampal-dependent learning and memory. To test this hypothesis, we irradiated 3-month-old male and female mice with a single, whole-body dose of 10, 50, or 100 cGy ^56^Fe ions (600 MeV, 181 keV/μm) at Brookhaven National Laboratory. Our data reveal complex, dynamic, time-dependent effects of HZE exposure on the hippocampus. Two months post exposure, neurogenesis, synaptic plasticity and learning were impaired compared to sham-irradiated, age-matched controls. By six months post-exposure, deficits in spatial learning were absent in irradiated mice, and synaptic potentiation was enhanced. Enhanced performance in spatial learning and facilitation of synaptic plasticity in irradiated mice persisted 12 months post-exposure, concomitant with a dramatic rebound in adult-born neurons. Synaptic plasticity and spatial learning remained enhanced 20 months post-exposure, indicating a life-long influence on plasticity and cognition from a single exposure to HZE in young adulthood. These findings suggest that GCR-exposure can persistently alter brain health and cognitive function during and after long-duration travel in deep space.

## Introduction

Beyond earth’s shielding magnetosphere, biological organisms encounter a harsh radiation environment due to high-energy galactic cosmic rays, solar particle events, and radiation belts. Exposure to large GCR fluxes composed of heavy, high-ionizing energy (HZE) particles with high linear energy transfer (LET) properties, such as charged ^56^Fe ions, induces a range of neurophysiological alterations in humans and experimental animals^1–4^. The prospect of encountering high-atomic number, high energy radiation during deep space missions, and its implications for astronaut health, spacecraft design, and mission success, has led researchers to focus on acute effects of GCR exposure. However, crewed missions to Mars and other deep-space destinations will require long-duration missions lasting many months and possibly years. Yet chronic and long-term effects of GCR exposure on brain structure and function are largely unexplored. These knowledge gaps pose potentially serious challenges to crew performance and brain health during and after long-duration missions in space.

Radiation has been shown to preferentially damage proliferating cells, likely due to the exposed nature of DNA during synthesis^5,6^. The pool of proliferative neural stem cells in the subgranular zone (SGZ) of the hippocampal dentate gyrus is acutely suppressed by exposure to HZE, observed as a reduction in 5-bromo-2′dioxyuridine (BrdU) incorporation^7–10^, reduction in number of doublecortin (DCX)-positive adult-born neurons^10–12^, and increase in apoptosis of neural precursor cells^13^. Taken together, these results confirm that the proliferative niche of neural stem cells in the dentate gyrus of the hippocampus is highly susceptible to HZE-induced damage. Consistent with these findings, HZE-exposure also impairs hippocampus-dependent spatial learning and memory in rodents measured in the Morris Water Maze^14,15^, Barnes Maze^16,17^, novel object recognition test^18,19^, and contextual fear conditioning^10,20^ up to three months post irradiation. Limoli and colleagues have shown that memory impairments in the novel object recognition task and fear conditioning in mice persist for 24 weeks after exposure to ^48^Ti or ^16^O ions^21^, and that the impairments in recognition memory persisted for a year after exposure to charge particles of ^4^He^22^. In another study, mice exposed to ^16^O-particle radiation were reported to demonstrate impaired novel object recognition, but not short-term memory when tested 9 months after exposure^23^. While these HZE exposure effects show complex dependencies on exposure parameters (i.e., HZE ion(s), dose, fluence, fractionation, field effects, as well as the model organism, strain, sex, age, etc.)^24^ the overarching conclusion of these studies is that HZE exposure is detrimental to adult hippocampal neurogenesis and hippocampus-dependent learning and memory in the acute and sub-acute period post-irradiation.

Persistent and possibly progressive long-term effects of GCR exposure represent a potentially serious challenge for long-duration crewed missions in deep-space, and to date, have been under-investigated. Therefore, we compared short and long-term effects of HZE exposure on the brain, focusing on the hippocampus as a critical brain region for declarative, contextual learning and memory. Dose-dependent, sex-dependent, and time-dependent characterization of the dynamics of hippocampal neurogenesis, hippocampal synaptic plasticity, and spatial learning and memory were used to address the following questions: How does an early, acute GCR insult to the hippocampus manifest later in life? Are GCR-induced damage and deficits involving the hippocampus permanent? Alternatively, might these effects trigger compensatory mechanisms that mitigate long-term cognitive dysfunction?

To address these questions, we exposed cohorts of adult male and female mice to an ^56^Fe (600 MeV, 181 keV/μm) source totaling 10, 50, or 100 cGy at a dose rate of 10 cGy/min using the particle beam line accelerator at the National Space Radiation Laboratory of Brookhaven National Laboratory, and compared them to age- and sex-matched, sham-irradiated controls at corresponding post-exposure time points (2, 6, 12, 20 months post-irradiation.) We found that adult hippocampal neurogenesis was suppressed two months post-irradiation, but rebounded to significantly above control levels by 12 months post-irradiation. Both long-term potentiation (LTP) of synaptic strength in the hippocampus and hippocampal-dependent spatial learning followed a similar pattern of months of impairment followed by enhancement. These findings suggest that compensatory mechanisms with life-long functional consequences are activated, and this may partially offset hippocampal dysfunction resulting from HZE exposure expected during long-duration deep-space travel.

## Results

### HZE exposure-induced suppression of adult neurogenesis in the dentate gyrus of the hippocampus is transient

**Figure 1:**
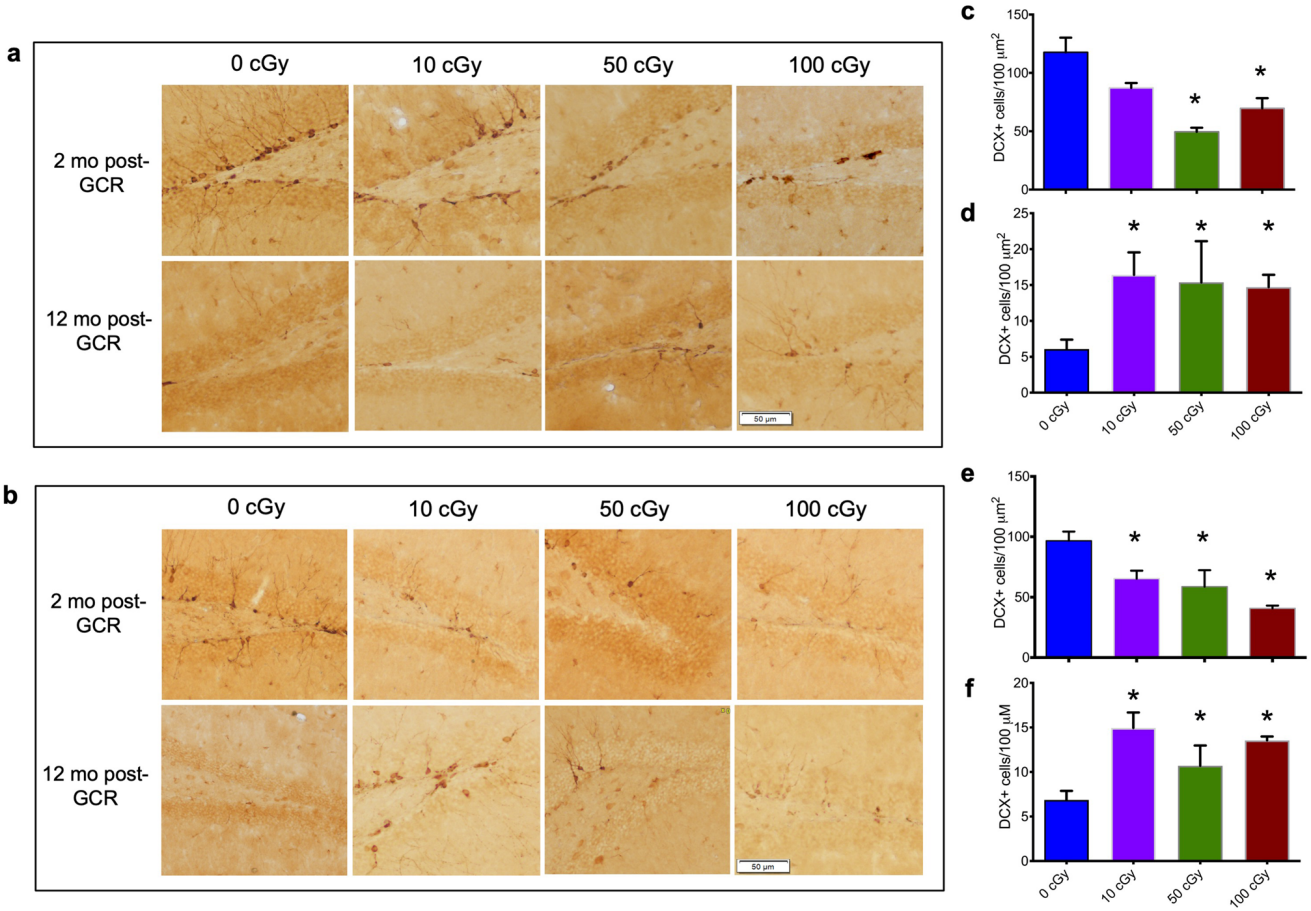
HZE exposure-induced suppression of adult neurogenesis in the dentate gyrus of the hippocampus is transient. Representative images (magnification 10 ×) of immunoreactivity of DCX + cells in coronal brain sections from male (**a**) and female (**b**) mice two months and 12 months post HZE exposure. Quantification of DCX + cell density in sections 2 months post exposure reveals deficits in both (**c**) male mice, One-way ANOVA, F(3,16) = 13.20, *P* = 0.0001, Tukey’s post hoc test: 0 cGy vs. 10 cGy, *P* = *0.070*; 0 cGy vs. 50 cGy, *P* < 0.0001; 0 cGy vs. 100 cGy, *P* = 0.003, 10 cGy vs. 50 cGy, *P* = 0.019, 10 cGy vs. 100 cGy, *P* = 0.427, 50 cGy vs. 100 cGy, *P* = 0.312] and (**e**) female mice, One-way ANOVA, F(3,16) = 15.08, *P* = 0.0001, Tukey’s post hoc test: 0 cGy vs. 10 cGy, *P* = 0.009; 0 cGy vs. 50 cGy, *P* = 0.002; 0 cGy vs. 100 cGy, *P* < 0.0001, 10 cGy vs. 50 cGy, *P* = 0.862, 10 cGy vs. 100 cGy, *P* = 0.047, 50 cGy vs. 100 cGy, *P* = 0.190, but elevated density of DCX + cells 12 months post radiation exposure in both (**d**) male mice, One-way ANOVA, F(3,14) = 4.85, *P* = 0.01, Tukey’s post hoc test: 0 cGy vs. 10 cGy, *P* = 0.025; 0 cGy vs. 50 cGy, *P* = 0.047; 0 cGy vs. 100 cGy, *P* = *0.046*, 10 cGy vs. 50 cGy, *P* = *0.988*, 10 cGy vs. 100 cGy, *P* = 0.947, 50 cGy vs. 100 cGy, *P* = 0.996, and **(f)** female mice One-way ANOVA, F(3,12) = 9.92, *P* = 0.001, Tukey’s post hoc test: 0 cGy vs. 10 cGy, *P* = 0.002; 0 cGy vs. 50 cGy, *P* = 0.018; 0 cGy vs. 100 cGy, *P* = 0.005, 10 cGy vs. 50 cGy, *P* = 0.123, 10 cGy vs. 100 cGy, *P* = 0.798, 50 cGy vs. 100 cGy, *P* = 0.362, compared to age-matched sham-irradiated controls (0 cGy) is shown in bar graphs. *P < 0.05 compared to 0 cGy. Data represents mean ± SEM. *n* = 5 mice per dose per time point, 6 sections per mouse.

Acute and short-term suppression of hippocampal neurogenesis after exposure to simulated GCR has been extensively studied^7,8,11–13,25^. To determine if GCR-induced suppression of hippocampal neurogenesis persists chronically, we evaluated the effects of three ^56^Fe exposure doses (10, 50, 100 cGy) on the population of immature, adult-born neurons, in male and female mice at 2 months and 12 months post-irradiation (Fig. 1a,b). At 2 months post-irradiation, we confirmed suppression of hippocampal neurogenesis compared to sham-irradiation in both males [Fig. 1c; One-way ANOVA, F(3,16) = 13.20, *P* = 0.0001] and females [Fig. 1e; One-way ANOVA, F(3,16) = 15.08, *P* = 0.0001], as indicated by a reduction in the number of cells expressing the immature neural marker, doublecortin (DCX). Further multiple comparison analysis revealed that even the lowest ^56^Fe exposure dose (10 cGy) significantly reduced the number of DCX-positive cells in female mice compared to controls (*P* = 0.009), but this dose only resulted in a moderate reduction in neurogenesis in male mice (*P* = 0.067). Fifty cGy exposure was the lowest dose which led to a significant reduction in DCX-positive cells in male mice at 2 months (*P* > 0.0001), suggesting sex-differences in predisposition to GCR-mediated insult. The magnitude of reduced neurogenesis was not significantly different between mice exposed to 50 cGy or 100 cGy, suggesting 50 cGy exposure is sufficient to induce a maximal reduction in neurogenesis in both male and female mice. Despite the reduction in newly born neurons 2 months post-exposure, the population of adult born neurons in cohorts evaluated for neurogenesis 12 months post-exposure significantly exceeded control levels in both male [Fig. 1d; One-way ANOVA, F(3,14) = 4.85, *P* = 0.01] and female mice [Fig. 1f; One-way ANOVA, F(3,12) = 9.92, *P* = 0.001]. It should be noted that the decline in the number of DCX + cells from 2 to 12 months in controls is consistent with normal age-related decline in neurogenesis^26^, therefore all comparisons were made between strictly age-matched cohorts. These findings indicate that exposure to simulated ^56^Fe GCR results in a decline in the population of newly born neurons at 2 months after irradiation significant and an unexpected rebound in neurogenesis 12 months post-exposure. This phenomenon, possibly resulting from compensatory or repair mechanisms which far outlast the direct effects of radiation itself, was observed even for the lowest ^56^Fe dose tested (10 cGy). Such dramatic, long-lasting effects are likely to influence synaptic transmission and plasticity in the hippocampus and other brain regions, and thereby influence hippocampal-dependent and independent cognitive function.

### Impairments in Schaffer collateral-CA1 LTP and spatial learning two months after exposure to ^56^Fe particle radiation

**Figure 2:**
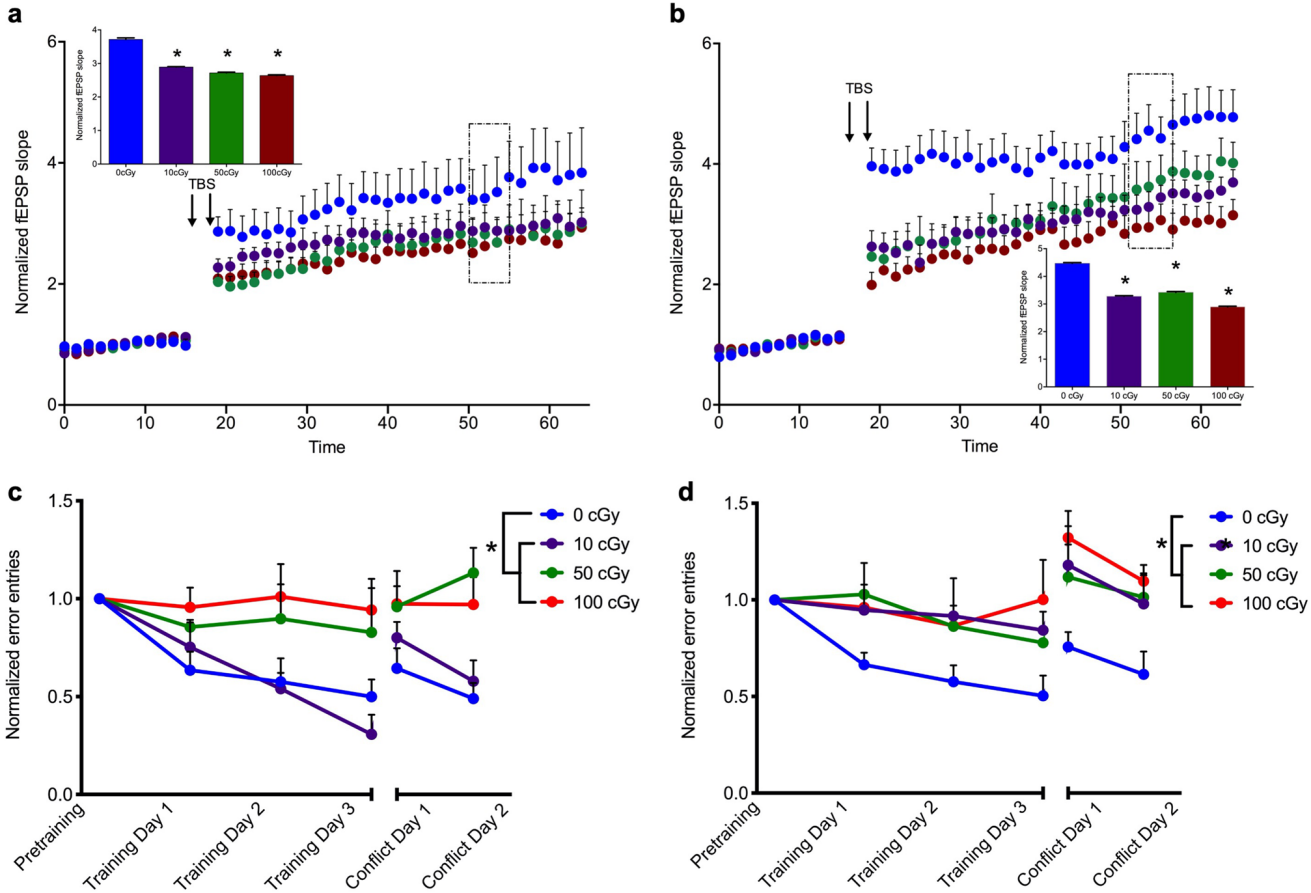
Impairments in Schaffer collateral-CA1 LTP and spatial learning two months after exposure to ^56^Fe particle radiation (**a**, **b**) Time course and magnitude (inset bar graph) of LTP in slices from mice exposed to 10 cGy, 50 cGy, or 100 cGy radiation, compared to sham-irradiated controls (0 cGy). After a 15 min baseline, LTP was elicited by two high-frequency TBS stimulus trains (arrows), and magnitude of LTP between 35 and 40 min post TBS (perforated box) was compared across doses in (**a**) male mice, One-way RM ANOVA, F(1.84,18.35) = 292.8, *P* < 0.0001, Tukey’s post hoc test: 0 cGy vs. 10 cGy, *P* < 0.0001; 0 cGy vs. 50 cGy, *P* < 0.0001; 0 cGy vs. 100 cGy, *P* < 0.0001, 10 cGy vs. 50 cGy, *P* = 0.00*8*, 10 cGy vs. 100 cGy, *P* < 0.0001, 50 cGy vs. 100 cGy, *P* = 0.041*,* and **(b)** female mice, One-way RM ANOVA, F(2.06,20.59) = 1169.0, *P* < 0.001, Tukey’s post hoc test: 0 cGy vs. 10 cGy, *P* < 0.0001; 0 cGy vs. 50 cGy, *P* < 0.001; 0 cGy vs. 100 cGy, *P* < 0.0001, 10 cGy vs. 50 cGy, *P* < 0.0001, 10 cGy vs. 100 cGy, *P* < 0.0001, 50 cGy vs. 100 cGy, *P* < 0.0001*.* *P < 0.05 compared to 0 cGy, n = 12–16 slices per dose per sex. (**c**, **d**) Learning curves, including pretraining, training days 1–3, and conflict training days 1–2, are shown for (**c**) male mice, Two-way RM ANOVA, *dose*: F(3,16) = 4.51, *P* = 0.017; *trial*: F(3.59,57.51) = 7.22, *P* = 0.002; *interaction*: F(15,80) = 2.29, *P* = *0.*009, Tukey’s post hoc test: Conflict Day 2, 0 cGy vs. 50 cGy, *P* = 0.01, 0 cGy vs. 100 cGy, *P* = 0.043 and (**d**) female mice, Two-way RM ANOVA, *dose:* F(3,16) = 3.58, *P* = 0.037; *trial:* F(4.29,68.72) = 6.86, *P* < 0.001; *interaction*: F(15,80) = 1.15, *P* = 0.32, Tukey’s post hoc test: Training Day 1, 0 cGy vs. 100 cGy, P = 0.043, Conflict Day 1, 0 cGy vs. 100 cGy, *P* = 0.027*,* Conflict Day 2, 0 cGy vs. 100 cGy, *P* = 0.029 as a function of normalized number of entries into the stationary shock zone (Errors). *P < 0.05, n = 5 mice per dose per sex. Each point represents mean errors normalized to pre-training entries ± SEM.

Parallel to studies on the short-term effects of HZE exposure on hippocampal neurogenesis, several studies have shown impairments in synaptic transmission and plasticity at multiple hippocampal synapses^22,27–30^. To investigate the time-dependent effects of exposure to GCR-relevant particles on synaptic plasticity, we first measured the magnitude of LTP of stimulus-evoked synaptic transmission at Schaffer collateral synapses in the CA1 subfield of the hippocampus two months after exposure to ^56^Fe. In this two months post-exposure group, radiation treatment was associated with a significant impairment in the magnitude of LTP elicited, determined by the average slope of the field excitatory postsynaptic potential (fEPSP) 35–40 min after theta-burst stimulation (TBS) normalized to the average fEPSP during a 15 min pre-TBS baseline, in both male [Fig. 2a; One-way RM ANOVA, F(1.84,18.35) = 292.8, *P* < 0.001] and female mice [Fig. 2b; One-way RM ANOVA, F(2.06,20.59) = 1169.0, *P* < 0.001]. Multiple comparison analysis revealed a significant dose effect in both male (0 cGy vs. 10 cGy, *P* < 0.001; 10 cGy vs. 50 cGy, *P* = 0.001; 50 cGy vs. 100 cGy, *P* = 0.04) and female (0 cGy vs. 10 cGy, *P* < 0.001; 10 cGy vs. 50 cGy, *P* < 0.001; 50 cGy vs. 100 cGy, *P* < 0.001) cohorts.

Since the acute and short-term alterations in neurophysiology have also been strongly associated with deficits in spatial learning and memory^10,14,15,17,18,20^ following exposure to HZE ions, we next sought to investigate whether the impairments in LTP we observed two months post-exposure were associated with alterations in hippocampus-dependent spatial learning and memory. To test this, we utilized the Active Place Avoidance task, in which mice learn to avoid a stationary shock zone on a rotating platform using stationary cues in the arena^31^. Analysis of learning curves generated by the number of entries into the shock zone (Errors) normalized to the number of entries to the shock zone during “pre-training” when the shock was disabled, revealed a statistically significant group effect in both male [Fig. 2c; Two-way RM ANOVA, *dose*: F(3,16) = 4.51, *P* = 0.017; *trial*: F(3.59,57.51) = 7.22, *P* = *0.002*; *interaction*: F(15,80) = 2.29, *P* = 0.009] and female [Fig. 2d; Two-way RM ANOVA, *dose:* F(3,16) = 3.58, *P* = 0.037; *trial:* F(4.29,68.72) = 6.86, *P* < 0.001; *interaction*: F(15,80) = 1.15, *P* = 0.32] cohorts of irradiated mice two months post-exposure, compared to sham-irradiated controls. Further multiple comparison analyses showed that while learning impairment was not dose-dependent, conflict learning, or memory discrimination, during which the location of the shock zone is changed, was significantly impaired by both 50 cGy and 100 cGy (Fig. 2c; *P* = 0.01 and *P* = 0.043, respectively) but not by 10 cGy (*P* = 0.843) in male mice. Learning flexibility was also impaired in female mice two months post-exposure, significantly by 100 cGy exposure (Fig. 2d; *P* = 0.029).

In this cohort of mice at two months post-exposure, and in cohorts of mice at following time points, the OptoMotry test for visual acuity (Supplementary Fig. S1A–D), elevated platform test for anxiety-related behavior (Supplementary Fig. S2A–D), and Open Field locomotion testing (Supplementary Fig. S3A–D) did not reveal differences following any dose of ^56^Fe particle exposure, ruling out the possibility that the observed differences in performance in spatial learning tasks were influenced by visual or motor impairment, or differing levels of anxiety.

### Enhancement in Schaffer collateral-CA1 LTP and recovery of spatial learning deficits six months after exposure to ^56^Fe particle radiation

**Figure 3:**
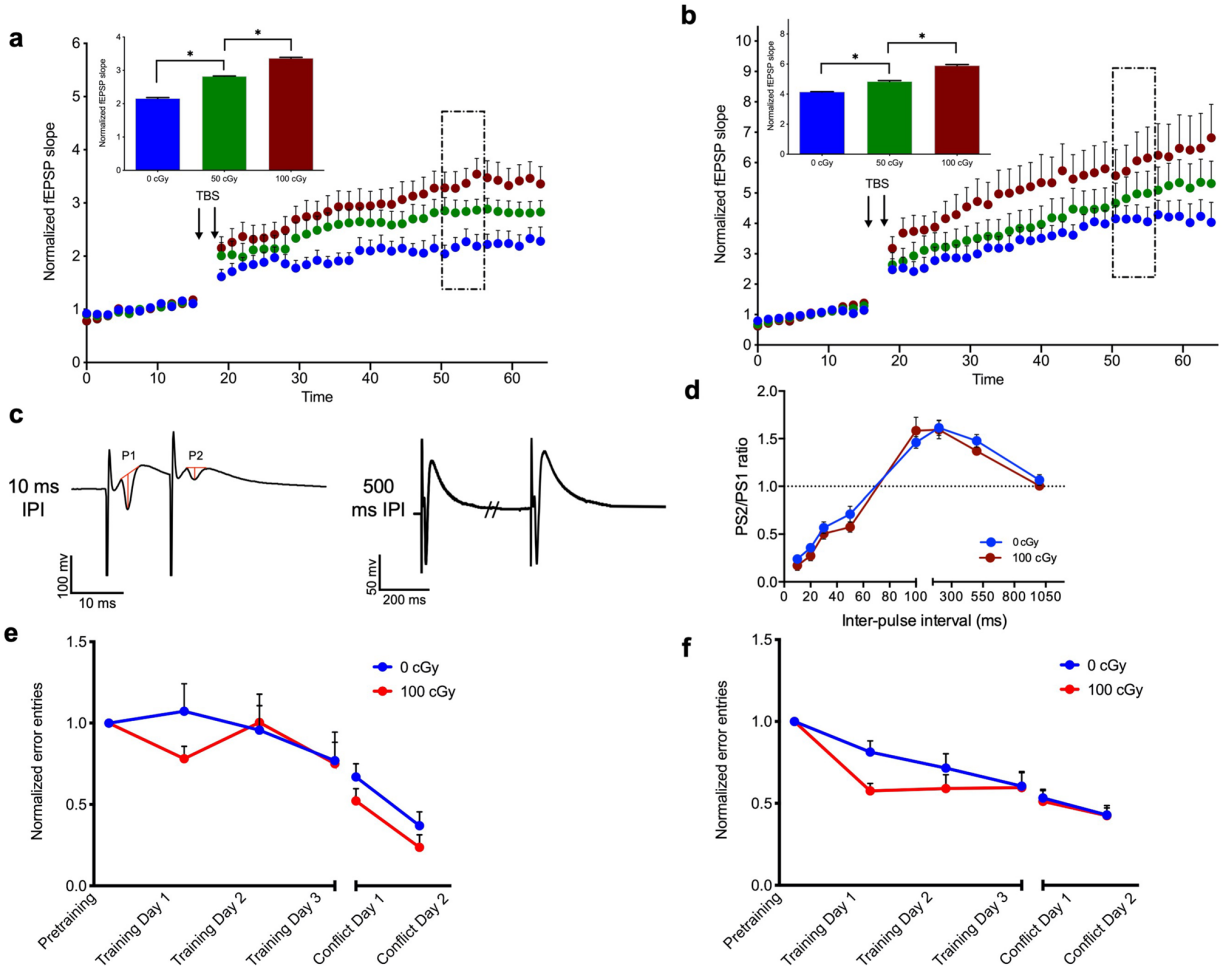
Enhancement in Schaffer collateral-CA1 LTP and recovery of spatial learning deficits six months after exposure to ^56^Fe particle radiation (**a**, **b**) Time course and magnitude (inset bar graph) of LTP in slices from mice exposed to 10 cGy, 50 cGy, or 100 cGy radiation, compared to sham-irradiated controls (0 cGy). After a 15 min baseline, LTP was elicited by two high-frequency TBS stimulus trains (arrows), and magnitude of LTP between 35–40 min post TBS (perforated box) was compared across doses in (**a**) male mice, One-way RM ANOVA, F(1.95,19.45) = 820.8, *P* < 0.0001, Tukey’s post hoc test: 0 cGy vs. 50 cGy, *P* < 0.0001; 0 cGy vs. 100 cGy, *P* < 0.0001, 50 cGy vs. 100 cGy, *P* < 0.0001 and (**b**) female mice, One-way RM ANOVA, F(1.30,12.95) = 393.0, P < 0.0001, Tukey’s post hoc test: 0 cGy vs. 50 cGy, *P* < 0.0001; 0 cGy vs. 100 cGy, *P* < 0.0001, 50 cGy vs. 100 cGy, *P* < 0.0001. *P < 0.05 compared to 0 cGy, n = 12–16 slices per dose per sex. **(c)** Representative paired-pulse responses of population compound action potentials elicited in slices from a male sham-irradiated mouse, taken at 10 and 500 ms inter-stimulus interval. (**d**) Mean paired-pulse profiles across inter-stimulus intervals in all slices from control (0 cGy) and irradiated (100 cGy) mice. Each point represents mean ± SEM. n = 12–16 slices per dose per sex. (**e**, **f**) Learning curves, including pretraining, training days 1–3, and conflict training days 1–2, are shown for **(e)** male mice, Two-way RM ANOVA, *dose*: F(1,8) = 0.59, *P* = 0.59, *trial*: F(2.43,19.43) = 18.88, *P* < 0.0001; *interaction*: F(5,40) = 0.993, *P* = *0.434*, (Tukey’s post hoc test did not reveal significant within treatment effect) and (**f**) female mice, Two-way RM ANOVA, *dose*: F(1,8) = 0.99, *P* = 0.99; *trial*: F(1.96,15.66) = 31.54, *P* < *0*.0001; *interaction*: F(5,40) = 1.896, *P* = 0.117, (Tukey’s post hoc test did not reveal significant within treatment effect), as a function of normalized number of entries into the stationary shock zone (Errors). n = 5 mice per dose per sex. Each point represents mean errors normalized to pre-training entries ± SEM.

Having confirmed previous findings that HZE exposure results in deficits in synaptic plasticity acutely after exposure, we tested LTP at Schaffer-CA1 synapses six months post-exposure. Six months post ^56^Fe exposure, the normalized magnitude of LTP between 35 and 40 min post TBS exhibited dose-dependent *enhancement* in both the male cohort [Fig. 3a; One-way RM ANOVA, F(1.95,19.45) = 820.8, *P* < 0.0001; 0 cGy vs. 50 cGy, *P* < 0.001; 50 cGy vs. 100 cGy, *P* < 0.001] and in the female cohort [Fig. 3b; One-way RM ANOVA, F(1.30,12.95) = 393.0, P < 0.0001; 0 cGy vs. 50 cGy, *P* < 0.001; 50 cGy vs. 100 cGy, *P* < 0.001]. To address whether enhanced LTP in CA1 could be a result of decreased GABAergic synaptic inhibition, we compared paired-pulse inhibition/facilitation profiles of evoked population spikes in slices from male mice 6 months post-exposure (Fig. 3c,d). Differences in paired-pulse response profiles between doses would suggest differences in inhibitory drive on glutamatergic neurons. We observed no significant shift in paired-pulse inhibition or facilitation at any tested inter-pulse-interval (IPI) between 0 and 100 cGy at six months post-HZE exposure. This suggests that enhanced LTP is not explained by decreased GABAergic drive.

Given the co-occurrence of deficits in spatial learning and synaptic plasticity two months post exposure, we next considered if restoration of plasticity six months post exposure was reflected in spatial learning. The active avoidance task was again employed to test learning and memory flexibility. Analysis of the learning curves generated by number of error-entries into the shock zone revealed that the impairments in spatial learning observed two months post-exposure were absent at 6 months post-exposure in both male [Fig. 3e; Two-way RM ANOVA, *dose*: F(1,8) = 0.59, *P* = 0.59, *trial*: F(2.43,19.43) = 18.88, *P* < 0.001; *interaction*: F(5,40) = 0.993, *P* = 0.434] and female cohorts [Fig. 3f; Two-way RM ANOVA, *dose*: F(1,8) = 0.99, *P* = 0.99; *trial*: F(1.96,15.66) = 31.54, *P* < 0.001; *interaction*: F(5,40) = 1.896, *P* = 0.117] exposed to 100 cGy ^56^Fe particles, compared to age-matched controls. Multiple comparison analysis revealed that neither males nor females 6 months post-exposure performed significantly differently than their age-matched controls in conflict learning (*P* = 0.86 and *P* > 0.99, respectively), a deficit that was present 2 months post-exposure. To examine if the rebound in LTP and behavioral deficits were persistent, we next tested male and female mice 12 months post-exposure.

### Enhancements in Schaffer collateral-CA1 LTP and spatial learning 12 months after exposure to ^56^Fe particle radiation

**Figure 4:**
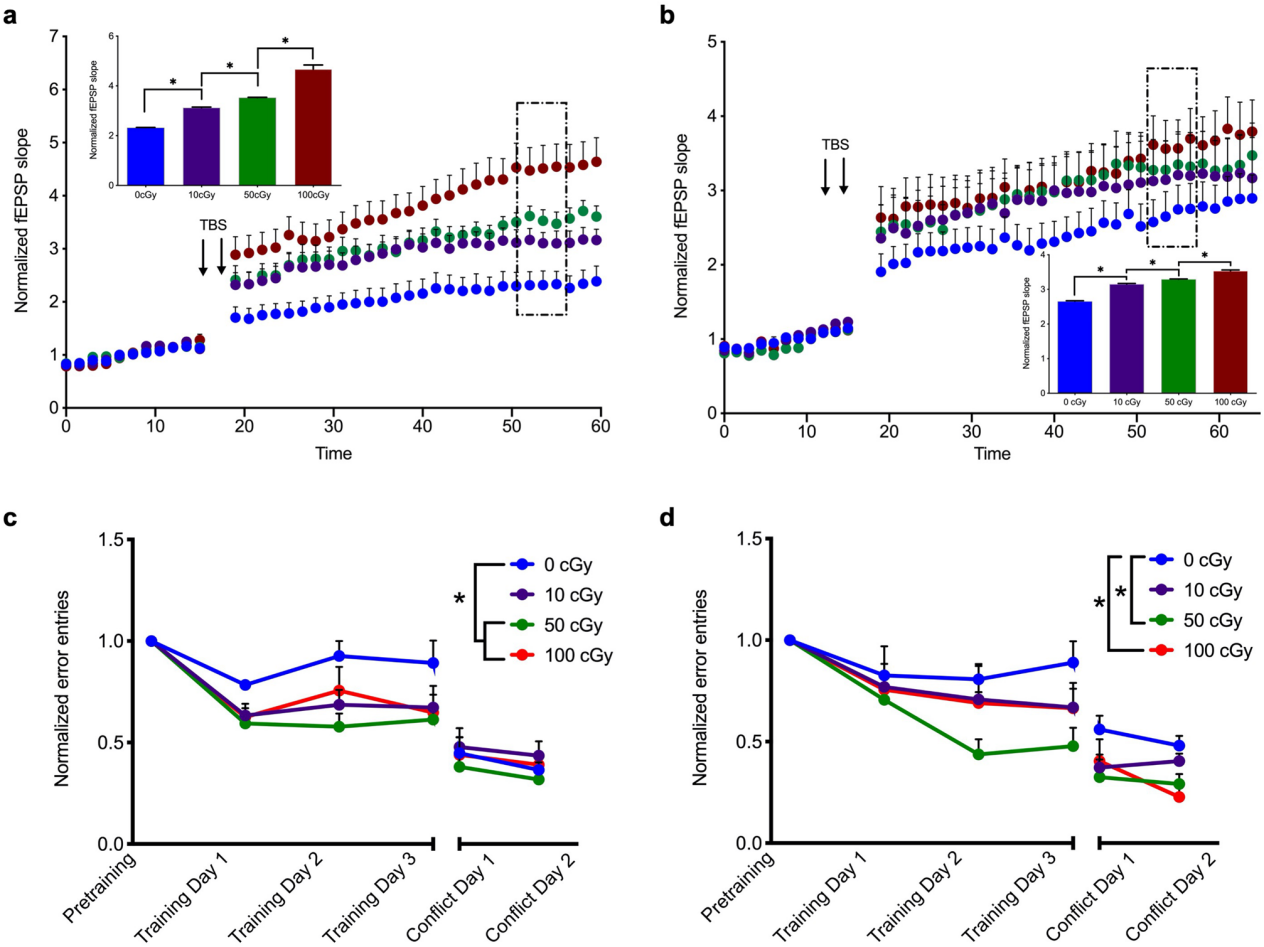
Enhancements in Schaffer collateral-CA1 LTP and spatial learning 12 months after exposure to ^56^Fe particle radiation (**a**, **b**) Time course and magnitude (inset bar graph) of LTP in slices from mice exposed to 10 cGy, 50 cGy, or 100 cGy radiation, compared to sham-irradiated controls (0 cGy). After a 15 min baseline, LTP was elicited by two high-frequency TBS stimulus trains (arrows), and magnitude of LTP between 35 and 40 min post TBS (perforated box) was compared across doses in (**a**) male mice, One-way RM ANOVA, F(1.73,17.31) = 3137.0, *P* < 0.001, Tukey’s post hoc test: 0 cGy vs. 10 cGy, *P* < 0.0001; 0 cGy vs. 50 cGy, *P* < 0.0001; 0 cGy vs. 100 cGy, *P* < 0.0001, 10 cGy vs. 50 cGy, *P* < 0.0001, 10 cGy vs. 100 cGy, *P* < *0.0001*, 50 cGy vs. 100 cGy, *P* < 0.0001 and (**b**) female mice, One-way RM ANOVA, F(2.48,24.77) = 449.1, *P* < 0.0001, Tukey’s post hoc test: 0 cGy vs. 10 cGy, *P* < 0.0001; 0 cGy vs. 50 cGy, *P* < 0.000*1*; 0 cGy vs. 100 cGy, *P* < 0.0001, 10 cGy vs. 50 cGy, *P* < 0.0001, 10 cGy vs. 100 cGy, *P* < 0.0001, 50 cGy vs. 100 cGy, *P* < 0.0001. *P < 0.05 compared to 0 cGy, n = 12–16 slices per dose per sex. (**c**, **d**) Learning curves, including pretraining, training days 1–3, and conflict training days 1–2, are shown for **(c)** male mice Two-way RM ANOVA, *dose*: F(3,16) = 2.78, *P* = 0.08; *trial*: F(1.933,30.93) = 54.98, *P* < 0.0001; *interaction*: F(15,80) = 1.31, *P* = *0.21*, Tukey’s post hoc test: Training Day 1, 0 cGy vs. 50 cGy, *P* = 0.008, 0 cGy vs. 100 cGy, *P* = 0.045, Training Day 2, 0 cGy vs. 50 cGy, *P* = 0.019 and **(d)** female mice Two-way RM ANOVA, *dose*: F(3,16) = 2.57, *P* = 0.09; *trial*: F(2.445,39.12) = 47.93, *P* < 0.0001; *interaction:* F(15,80) = 1.23, *P* = 0.266, Tukey’s post hoc test: Training Day 2, 0 cGy vs. 50 cGy, *P* = 0.021, Training Day 3, 0 cGy vs. 50 cGy, *P* = 0.044, Conflict Day 2, 0 cGy vs. 100 cGy, *P* = 0.038, as a function of normalized number of entries into the stationary shock zone (Errors). *P < 0.05, n = 5 mice per dose per sex. Each point represents mean errors normalized to pre-training entries ± SEM.

Given the enhancement of LTP and restoration of spatial learning ability six months post exposure to ^56^Fe, we next examined these two measures at 12 months post-exposure, by which point we found neurogenesis exceeded age-matched control levels (Fig. 1). As observed at six months post-exposure, the magnitude of LTP elicited 12 months post-exposure was significantly greater in a dose dependent manner in both male [Fig. 4a; One-way RM ANOVA, F(1.73,17.31) = 3137.0, *P* < 0.0001; 0 cGy vs. 50 cGy, *P* < 0.0001; 50 cGy vs. 100 cGy, *P* < 0.0001] and female mice [Fig. 4b; One-way RM ANOVA, F(2.48,24.77) = 449.1, *P* < 0.0001; 0 cGy vs. 50 cGy, *P* < 0.0001; 50 cGy vs. 100 cGy, *P* < 0.0001].

Based on our findings of enhanced hippocampal neurogenesis and synaptic plasticity 12 months post-exposure, we investigated whether these enhancements were correlated with performance in the active avoidance task. Remarkably, we measured a persistent though modest enhancement in learning performance 12 months after exposure to ^56^Fe particles (Fig. 4c,d). While overall learning curves revealed only a modest group effect of dose on learning for males [Fig. 4c; Two-way RM ANOVA, *dose*: F(3,16) = 2.78, *P* = *0.08*; *trial*: F(1.933,30.93) = 54.98, *P* < 0.001; *interaction*: F(15,80) = 1.31, *P* = 0.217] and females [Fig. 4d; Two-way RM ANOVA, *dose*: F(3,16) = 2.57, *P* = 0.09; *trial*: F(2.445,39.12) = 47.93, *P* < 0.001; *interaction:* F(15,80) = 1.23, *P* = 0.266], multiple comparison analysis revealed that in male mice, 50 cGy and 100 cGy exposure to ^56^Fe significantly enhanced learning on Day 1 (Fig. 4c; *P* = 0.007 and *P* = 0.045, respectively) when compared to age-matched controls. Female mice, while modestly enhanced in learning performance by all doses, learned significantly better on Days 2 and 3 of training (Fig. 4d; *P* = 0.02 and *P* = 0.04, respectively) if exposed to 50 cGy. Further, while learning flexibility did not differ significantly among doses and controls in male mice, female mice exposed to 100 cGy were able to re-learn a new shock position significantly better than controls (Fig. 4d; *P* = 0.037). To determine whether the chronic facilitation in synaptic plasticity and enhancement of spatial learning ability following exposure to charged ^56^Fe particles manifest persistently, we conducted further studies at our final time point, 20 months post-exposure.

### Enhancements in Schaffer collateral-CA1 LTP and spatial learning 20 months after exposure to ^56^Fe particle radiation

**Figure 5:**
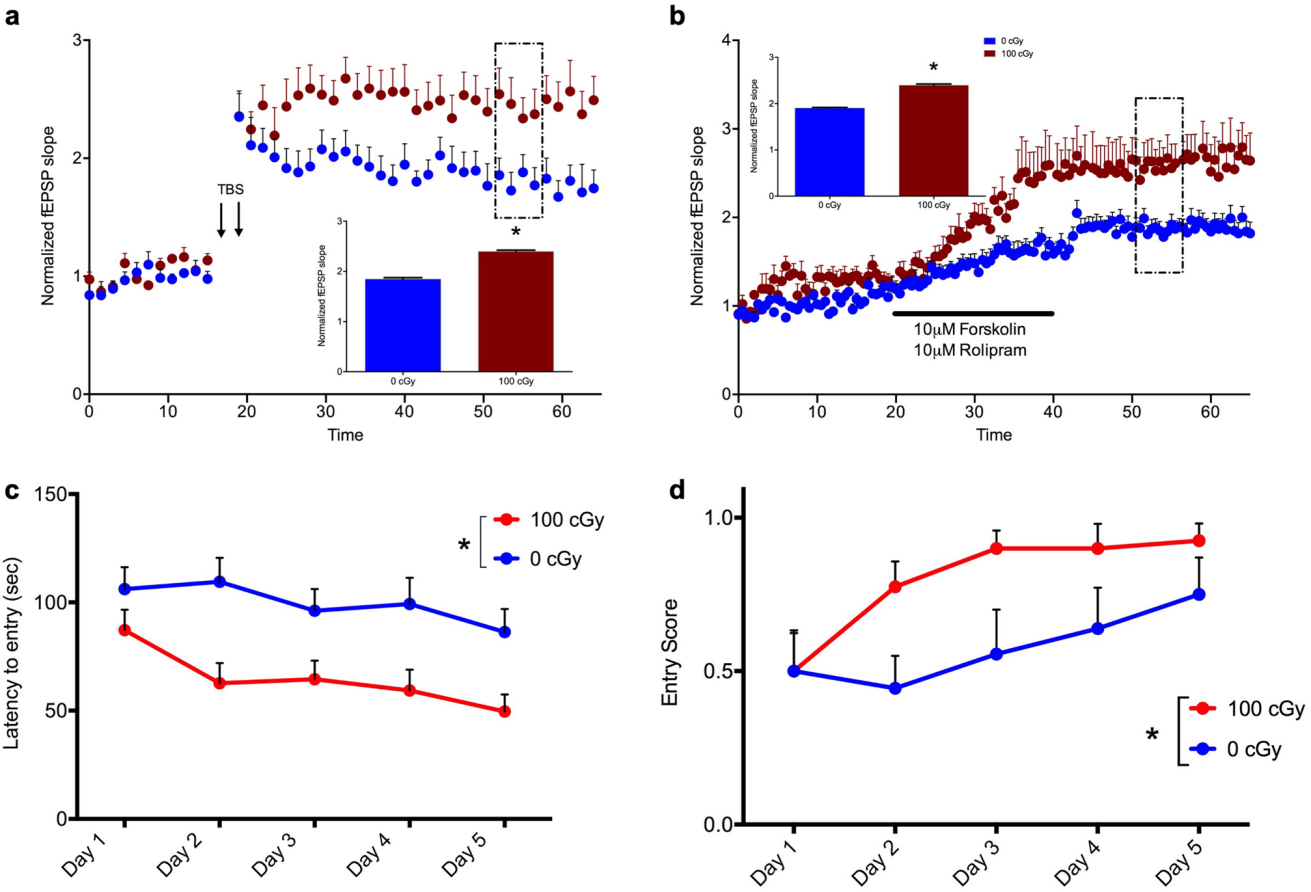
Enhancements in Schaffer collateral-CA1 LTP and spatial learning 20 months after exposure to ^56^Fe particle radiation (**a**) Time course and magnitude (inset bar graph) of stimulus-evoked LTP in slices from male mice exposed to 100 cGy radiation, compared to sham-irradiated controls (0 cGy). After a 15 min baseline, LTP was elicited by two TBS stimulus trains (arrows), and the magnitude of LTP between 35–40 min post TBS or bath application (perforated box) compared across doses. *P < 0.0001, Two-tailed t test, n = 12–16 slices per dose. (**b**) Time course and magnitude (inset bar graph) of chemically-evoked LTP elicited by bath application (bar) of 10 µM forskolin plus 10 µM rolipram to slices from male mice exposed to 100 cGy radiation compared to sham-irradiated controls (0 cGy). Each point represents mean ± SEM. *P < 0.0001, Two-tailed t test, n = 12–16 slices per dose. (**c**, **d**) Learning curves depicting (**c**) latency for entry into an escape box in the Barnes Maze, Two-way RM ANOVA, *dose*: F(1,90) = 30.73, *P* < 0.0001; *trial*: F(4,90) = 2.22, *P* = 0.073; *interaction*: F(4,90) = 0.553, *P* = 0.698, Tukey’s post hoc test: Day 2, 0 cGy vs. 100 cGy, *P* = 0.006, Day 4, 0 cGy vs. 100 cGy, *P* = 0.027, or **(d)** the fraction of successful trials per day, Two-way RM ANOVA, *dose:* F(1,90) = 10.51, *P* = 0.002, *trial*: F(4,90) = 3.06, *P* = 0.020; *interaction*: F(4,90) = 0.849, *P* = 0.497, (Tukey’s post hoc test did not reveal significant within treatment effect), are shown for male mice exposed to 100 cGy radiation compared to sham-irradiated controls (0 cGy). Each point represents mean latency ± sem. *P < 0.05, n = 10 mice per dose.

Because our data showed persistent compensatory enhancement in synaptic plasticity and spatial learning up to 12 months post-exposure to ^56^Fe, we addressed whether this enhancement was life-long, up to 20 months post-exposure in a 23-month-old cohort (Fig. 5). In slices from male mice exposed to 100 cGy charged ^56^Fe particles 20 months earlier, the magnitude of TBS-induced LTP was significantly enhanced (Fig. 5a; Two-tailed t test, *P* < 0.001) compared to sham-irradiated, age-matched controls. Since the paired-pulse profiles measured 6 months post-exposure argue against changes in inhibitory input as an explanation for enhanced LTP, we sought to determine whether synaptic plasticity signaling cascades downstream of synaptic activation are persistently altered. We thus induced LTP chemically by bath application of the adenylate cyclase stimulator forskolin (10 μM) and phosphodiesterase IV inhibitor rolipram (10 μM) to increase intracellular concentrations of cAMP and elicit chemical, stimulus independent LTP (Fig. 5b). Chemical LTP after a 20-min bath application of rolipram and forskolin was significantly enhanced (Fig. 5b; Two-tailed t test, *P* < 0.0001) by a similar magnitude to what we observed following stimulus-induced LTP (Fig. 5a) 20 months post-exposure to 100 cGy compared to controls.

Finally, to determine whether this consistent and persistent enhancement in LTP of synaptic strength was correlated with enhanced spatial learning and memory, we utilized the Barnes Maze, a spatial learning task more adaptable to geriatric mice than the Active Avoidance task. Male mice tested 20 months post-exposure to charged ^56^Fe particles learned the location of an escape box significantly faster than their age-matched control counterparts [Fig. 5c; Two-way RM ANOVA, *dose*: F(1,90) = 30.73, *P* < 0.001; *trial*: F(4,90) = 2.22, *P* = 0.073; *interaction*: F(4,90) = 0.553, *P* = 0.698], and this performance advantage was significantly pronounced on Day 2 of learning (P = 0.04). Scored entry averages also showed significant enhancement in learning by day two [Fig. 5d; Two-way RM ANOVA, *dose:* F(1,90) = 10.51, *P* = 0.002, *trial*: F(4,90) = 3.06, *P* = 0.020; *interaction*: F(4,90) = 0.849, *P* = 0.497].

## Discussion

A critical limiting factor for human exploration of Mars and other extra-orbital destinations is the prospect of prolonged exposure to a harsh radiation environment due to high-energy galactic cosmic rays (GCRs), solar particle events, and radiation belts. The likelihood of experiencing high-atomic number, high energy (HZE) radiation, such as charged ^56^Fe particles during deep space missions, and its implications for astronaut health, spacecraft design, and mission success, has compelled researchers to focus on immediate to short-term effects of exposure on cognitive performance^24^. Evidence put forth strongly suggests multiple mechanisms of neural plasticity are impaired following exposure to HZE ions. One group employed a variety of charged particle sources to characterized neuronal effects, and found decreased dendritic complexity and spine density up to one month post exposure^29,30^, as well as increased cannabinoid type 1 receptor (CB1R)-dependent tonic GABA release onto hippocampal pyramidal cells five weeks post exposure^32^. Another study intriguingly found enhancement in Schaffer collateral-CA1 LTP three months after nine-week-old mice were exposed to ^28^Si particles, but this relative enhancement was attenuated by cognitive testing, suggesting a shift in ceiling limits of LTP^33^. The longest post-exposure measurement time points examined thus far revealed persistence of altered synaptic transmission and cognitive deficits up to one year after exposure^21–23^, a phenomenon now termed “space brain”^34^, and raises concern about the long-term safety of deep space travel for humans.

Some of the confounding factors making it difficult to reach consensus on the neurobiological effects of HZE exposure include variability in the model organism (mouse, rat, or rabbit), strain, sex, age of subjects at time of radiation exposure, and age at the measurement time point. Exacerbating the issue, the ion species chosen to constitute simulated GCRs, their fluence, energies, dose rates, and total doses delivered vary between labs and experiments. Nevertheless, a common and general trend can be extrapolated: HZE radiation, at least initially, potently suppresses adult hippocampal neurogenesis, impairs performance in hippocampus-dependent learning and memory tasks, and alters expression of synaptic plasticity in the hippocampus.

While understanding the short-term effects of HZE exposure is critical to preventing or mitigating damage, chronic effects of HZE exposure have largely remained unexplored, and are increasingly relevant for long-duration, deep space travel as proposed for a crewed mission to Mars^35–37^. Therefore, we characterized the effects of HZE exposure on the brain, particularly the hippocampus, in mice at several extended time points post-exposure to three different doses of ^56^Fe radiation. Dose-dependent, sex dependent, and time-dependent characterization of the dynamics of hippocampal neurogenesis, hippocampal synaptic plasticity, and spatial learning and memory was used to address these questions: (1) How does an early, acute insult to the hippocampus manifest later in life? (2) Is radiation-induced damage to the hippocampus persistent or compensated for by uncharacterized mechanisms, and (3) How is this reflected in cognitive performance later in life?

To address these questions, we exposed several cohorts of mice to three doses of charged ^56^Fe particles and compared them to age- and sex-matched sham-irradiated controls at corresponding post-exposure time points (2, 6, 12, and 20 months post-exposure.) Our early post-exposure timepoint (2 months) confirmed previous reports^7,8,10–12,25^ that hippocampal neurogenesis, assayed by measuring the number of immature adult-born neurons, was suppressed for at least two months post-exposure. Importantly, we found that the lowest dose of exposure significantly impaired neurogenesis in females, but only moderately in males, suggesting female mice may be more prone to HZE-induced insult to neurogenesis. Given that male rodents have been shown to develop greater maladaptive responses to HZE-exposure than females^38^ and are generally more vulnerable to brain injury than females^39^, our results showing higher sensitivity in females is unexpected and may be reflective of a lower rate of baseline neurogenesis in female rodents^40,41^, which is also evident in our data. Remarkably though, the population of newly born neurons in both female and male mice, regardless of radiation dose, rebounded to significantly *higher* levels than sham-irradiated control levels when assayed later, at 12 months post-exposure. To our knowledge, this is the first demonstration of such a rebound phenomenon in the neurogenic capacity of the dentate gyrus following insult and suggests the presence of compensatory or repair mechanisms which far outlast the direct effects of radiation itself. Dose-dependent recovery of neurogenesis 270 days after exposure as high as 50 cGy Fe ions has been predicted with mathematic modeling^42^, suggesting neurogenesis recovers faster than our six-month post-exposure timepoint for our lower dose exposures.

DCX expression, which we used as a proxy for neurogenesis, labels neurons in an immature state. Whether changes in the number of DCX-expressing cells reflects changes in proliferation or survival of immature neurons cannot be inferred from our data. Regardless, such dramatic, long-lasting effects on the population of immature neurons are likely to contribute to other forms of hippocampal plasticity and may have a lasting effect on general cognitive ability, learning and memory.

Our early post-exposure timepoint also verified previous reports of impaired neural transmission and synaptic plasticity. However, the irradiation-induced impairment in synaptic plasticity we observed 2 months post-exposure was absent by 6 months post-exposure. Instead, the magnitude of LTP elicited in slices from irradiated male and female mice was dose-dependently *enhanced* at 6, 12, and 20 months post-exposure. Our data showing unchanged paired-pulse facilitation/depression profiles 6 months post-exposure suggest changes in synaptic inhibitory drive are unlikely to underly the enhancement in LTP. Rather, we found that cAMP-induced chemical LTP, which is elicited independent of synaptic activation, was also enhanced, suggesting that compensatory mechanisms influenced neuronal plasticity *downstream* of glutamate receptors, including N-methyl-D-aspartate receptor activation, and included persistent and intrinsic shifts in biochemical mechanisms underlying homeostatic plasticity.

Finally, consistent with the time-dependent shift we observed, from impairment to facilitation in synaptic plasticity in the hippocampus, our data reveal that hippocampus-dependent spatial learning was impaired 2 months post-exposure to ^56^Fe, but learning performance had recovered by 6 months post-exposure in both male and female mice. Intriguingly, at 12 months post exposure, both male and female mice exposed to the higher doses of irradiation outperformed sham-irradiated controls in learning acquisition and learning flexibility, in which they had to learn a new shock location. This facilitation in spatial learning remarkably persisted to 20 months post-exposure to 100 cGy charged ^56^Fe in a male cohort tested. While enhanced hippocampus-dependent cognitive performance at these later post-exposure timepoints was unexpected based on previous reports^21,22,43^, a recent study reports improved pattern separation in a location discrimination touchscreen task and improved performance in contextual fear conditioning-based pattern separation task in mature (6 month-old) C57Bl/6 mice exposed to ^56^Fe or ^28^Si and assayed two months later^44^. The findings in this study not only demonstrate that it is not universally true that HZE-particle exposure has a negative impact on high-level cognition, but in light of our data, brings into focus the distinction between age and time. It is possible that, after a certain age, rather after a set amount of time, compensatory mechanisms counteracting harmful effects of irradiation are engaged. Future experiments controlling for all variables except for age of exposure and time of behavioral testing will help to elucidate this intersect. Nevertheless, our findings again demonstrate dramatic, long-lasting effects of exposure to GCR-relevant HZE ions to the hippocampus and broader cognitive function.

Whether there is a causal relationship between post-exposure neurogenesis dynamics, LTP, and learning, or a correlation due to shared compensatory mechanisms, our demonstration that a single HZE exposure induces chronic and persistent alterations in the hippocampus is novel and has significant implications for long duration space missions. However, while we have shown that HZE exposure has a life-long impact the brain, these findings can only be interpreted to mean that HZE exposure can elicit very long-lasting changes that may include activation of compensatory mechanisms in response to the initial challenge, rather than that HZE exposure may be beneficial to the central nervous system. For example, while the eventual enhancement in neurogenesis and magnitude of LTP after exposure coincides with better performance in a spatial learning task, it is important to note that we have only tested one brain structure, one synapse, and only employed cognitive tests assaying aversion-driven spatial learning, dependent on both hippocampal and non-hippocampal structures. It is possible that our findings may not completely apply to other brain structures, synapses, or tests for cognition. In addition to studying other brain structures and behaviors in the C57Bl/6 mouse, more evidence needs to be collected from other mouse strains and animal species before fully extrapolating to humans. Mathematical modeling has predicted significant variability in rat strain-dependent effects of radiation exposure on neurogenesis^45^, emphasizing this need.

Finally, the human brain is far more structurally and organizationally complex than the rodent brain, and significant differences in responses to space radiation may well exist. Relative risk modeling^46^ to extrapolate risks to humans based on rodent studies has been used to attempt to take into account the cosmic environment, shielding options, and human tissue dynamics, predicting that a 1000-day mission to Mars poses only a modest relative risk for performance in the hippocampus-dependent Novel Object Recognition task^47^, though the clinical significance of this is unknown. This type of analysis has also revealed that the large absorbed doses (i.e., 100 cGy) used in animal studies such as this one may be larger than what is expected for an astronaut to encounter, and may therefore lead to overestimation of risk when extrapolating to humans. A limitation of the current study is the absence of lower-dose thresholding experiments (i.e., 1–5 cGy exposure) that may lend themselves more accurately to human extrapolation modeling. Still, no extent of mathematical modeling or animal experiments will completely predict how the human brain will respond to the deep-space environment, particularly extrapolating effects of a short-term, higher level exposure, to lower level, cumulative exposures lasting 1000 days or longer. Advances in non-invasive recording technology, such as EEG-based measurements of auditory-evoked LTP in awake humans^48^, will allow further insight into how the human brain responds, and perhaps compensates for, effects of GCR during long duration space missions. Until then, the data presented here provide the first insights into the very long-term range of regenerative capacity of the hippocampus post-exposure to HZE, how it may influence synaptic plasticity, and the behavioral implications for such life-long transformations.

## Materials and methods

All experiments were conducted in accordance with and under approved protocols from the Institutional Animal Care and Use Committees of New York Medical College (NYMC) and Brookhaven National Laboratory (BNL). This study was carried out in compliance with the ARRIVE guidelines.

### Animals

Male and Female C57Bl/6J mice were purchased from Charles River, Inc. (Wilmington, MA) and group housed (Brookhaven Laboratory Animal Facility, Brookhaven National Laboratory, Brookhaven, NY) four to five per cage and maintained at an ambient temperature of 22 ± 2 °C with a 12/12 light/dark cycle. Mice were provided with standard mouse chow and water ad libitum. Mice were exposed to simulated GCR beams (NASA Space Radiation Laboratory, NSRL, at Brookhaven National Laboratory) at three months of age. Mice at this age are sexually mature and attained adult body morphology (body wight, skull thickness) and major brain development milestones. One week after exposure, mice were transferred to animal housing facilities at New York Medical College (Valhalla, NY). After transfer, mice were allowed to acclimate to their housing facilities at NYMC and BNL for at least 1 week before irradiation or experimental testing. The presence of cataracts in one or both eyes served as an exclusion criterion for behavior testing.

### Radiation parameters

Mice were exposed to whole-body particle radiation from an ^56^Fe source (600 MeV, 181 keV/μm at 0, 10, 50, 100 cGy), with a dose rate of 10 cGy/min and beam uniformity of ± 2.5% using the particle beam line facilities at the NASA Space Radiation Laboratory (NSRL, Brookhaven National Laboratory). The proposed particle radiation parameters were selected in consultation with the sponsoring agency (NASA) and Dr. Eleanor Blakely, Ph.D., Senior Radiobiophysicist (Lawrence Berkeley National Laboratory, Berkeley, CA).

### Immunohistology

Mice were anesthetized with a ketamine (100 mg/kg)/Xylazine (10 mg/kg) mixture. After transcardial perfusion with phosphate-buffered saline (PBS) and 10% neutral buffered formalin (NBF), brains were harvested and allowed to fix overnight in 10% NBF at 4 °C. Brains were subsequently cryoprotected in 30% sucrose (wt/vol) in 0.1 M PBS and 0.1% sodium azide (NaN_3_; wt/vol), and sectioned coronally on a freezing microtome (Leica Biosystems, Nussloch, Germany). Nine serial sets of 30 μm-thick free-floating brain sections were stored in 0.1% NaN_3_ in 0.1 M PBS at 4 °C until processing. One series of sections was selected from each plate for immunostaining. Briefly, free floating sections were pretreated (to inhibit non-specific staining) with 5% normal donkey serum (NDS) in 0.3% Triton X-100 in 0.1 M PBS for one hour at room temperature. Sections were then incubated with a goat polyclonal antibody directed against doublecortin (C-18:sc-8066, Santa Cruz Biotechnology, Dallas, TX, dilution 1:100) in 5% NDS in 0.1 M PBS at 4 °C overnight. The following day, sections were incubated with biotinylated-donkey anti-goat secondary antibody (PA1-28,664, Invitrogen/Thermo Fisher Scientific, Waltham, MA, dilution 1:500) in 5% NDS for 60 min followed by 60 min in avidin–biotin complex (Vectastain Elite ABC-HRP Kit, PK-6100; Vector Laboratories, Burlingame, CA). A chromogenic color reaction using diaminobenzidine (Impact DAB Substrate, SK-4105, Vector Laboratories) was then used to distinguish DCX + cells. Slices corresponding to dorsal hippocampus (bregma: − 1.35–− 3.28, The Mouse Brain Atlas in Stereotaxic Coordinates, Third Edition, Elsevier) were mounted on slides before coverslipping.

### Cell quantification

Sections were imaged at 10 × magnification and DCX + cells were manually quantified in ImageJ (NIH) by an investigator blind to experimental conditions. Within the series of sections selected for each mouse (see “Immunohistology methods”), all immunoreactive cells throughout the SGZ and granule cell layers of both blades of the dentate gyrus were included. The SGZ was defined as a 30 μm band between the granule cell layer and hilus. The number of cells counted per slice was normalized to the area of the dentate gyrus, determined by drawing a border around the dentate and connecting the blades with a tangent. Five slides were examined and quantitated for each brain, each with five to six sections, were quantified and averaged.

### Behavioral tests

Behavior tests were used to evaluate learning, memory, anxiety and depression-like behaviors in mice at selected time points post-irradiation. The behavioral test battery included the Barnes maze for spatial learning, active avoidance for spatial learning and discrimination, open field exploration, elevated plus maze, and visual acuity as we have previously reported^49^.

*Open field test* (AnyMaze, Stoelting Co, Wood Dale, IL) was used to evaluate locomotor performance, behavior responsivity to a novel environment (neophobia), and anxiety (thigmotaxis) as previously reported^49,50^. Several parameters were measured during a 5 min exploration session, including total distance and time spent traveling (ambulation), as well as a comparison of time spent in the center versus periphery of the field. Additionally, we calculated the total number of entries into the central zone and relative time spent there as a measure of anxiety.

*Elevated plus maze (anxiety test)* was performed with a standard plus-shaped apparatus (Columbus Instruments, Columbus, OH) as we have previously reported^49,50^. During the 5 min task, mice explored the plus-shaped maze, consisting of two open (dimly lit) arms and two closed (dark) arms. Anxiety-related behavior was extrapolated by the percent of time spent in the open arms compared to the closed arms. Additional measurements during the 5 min test period include number of entries into each arm and time spent in the center, as previously reported^49^.

*Barnes Maze (hippocampal learning/memory)* was conducted using a 20-box apparatus with 900 lx surface light intensity. Training sessions were conducted across four training trials per day for 5 consecutive days. The order of testing of individual subjects was the same throughout daily sessions but randomized across the four test days for a total of 20 trials. A single 3 min habituation trial preceded the learning trials. To initiate learning, each mouse was placed in the middle of the maze and released. The position of test subjects was tracked (AnyMaze, Stoelting Co) while locating a single escape box placed at a constant position. Spatial learning was assisted by distal visual cues (high-contrast shapes and patterns on walls) that remained constant across test sessions. Latency to find the escape box, trajectory velocity to the escape box, and total trajectory distance was assessed and recorded for each trial using position tracking software (AnyMaze, Stoelting Co., Inc.). For some Barnes Maze analysis, ‘Entry Score’ was calculated as the average of successful (1) and unsuccessful (0) trials.

*Active avoidance* The active avoidance paradigm was used to test spatial memory and memory extinction as previously reported^49,50^. Mice were placed on a custom-built, circular (40 cm diameter) platform which rotates clockwise at a speed of 1.5 revolutions per minute, and trained to avoid a 60° shock zone, which was defined within a region of the rotating arena. Entrance into the shock zone results in a brief constant current foot-shock (500 ms, 0.5 mA) that is scrambled across pairs of rods. The intershock interval was 1.5 s. The position of the mouse was tracked by PC-based software that analyzed images from an overhead camera and delivered shocks appropriately (AnyMaze, Stoelting Co., Inc.). Pretraining and each training trial lasted 10 min, with an intertrial interval of at least 50 min. *Initial training* Mice were habituated to handling and the training environment, during which time the shock was turned off and mice permitted to walk freely on the rotating platform (pretraining). In the next trial, the programmable animal shocker source (Stoelting Co., Inc.) was turned on and animals were trained to avoid a stationary shock zone defined by distal visual cues within the room. *Conflict* Following initial training, a conflict variant of the task was deployed to test cognitive flexibility^31^. The location of the shock zone was moved 180° from its position during initial training, into the preferred region in which each mouse spent the most time during the preceding two training sessions. Avoidance of the new shock zone location required suppression of the conditioned responses associated with avoiding the initial location, as well as relearning to avoid the new location, incorporation of new information regarding the location of aversive stimuli, and acquisition of a contextually adapted avoidance response. Cognitive flexibility is required to segregate experiences associated with each shock zone and select between these two conflicting behaviors^31^.

*Visual acuity* Mice were habituated to the OptoMotry apparatus (CerebralMechanics, Inc., www.cerebralmechanics.com) for five days and placed in the center of a closed box formed by 4 computer monitors displaying vertical sine wave gratings to represent a virtual cylinder. The virtual grating can be made to “rotate” at 2 rpm in either the clockwise or counterclockwise direction that reliably elicits an opto-fixation reflex in mice^51^. An investigator blind to experimental group monitored head-tracking movements and increased the frequency of the gratings (cycles/degree) until no head-tracking movements were elicited. The highest reflex-eliciting grating frequency was taken as the threshold of visual acuity, in cycles per degree (cyc/deg) as previously reported^51^.

### Hippocampus slice electrophysiology

Electrophysiological field potential recordings from Schaffer collateral-CA1 synapses in in vitro hippocampal slices were performed using standard methods as described previously^52,53^. Mice were decapitated under deep isoflurane anesthesia, the brains quickly removed, hemisected, and cut with a vibratome (Leica model VT1200S) at a thickness of 350 µm. The tissue block was glued with cyanoacrylate adhesive to a stage immersed in ice-cold sucrose-based artificial cerebrospinal fluid (aCSF), in mM: 87 NaCl, 25 NaHCO_3_, 25 glucose, 75 sucrose, 2.5 KCl, 1.25 NaH_2_PO_4_, 0.5 CaCl_2_ and 7 MgCl_2_ (equilibrated with 95% O_2_/5% CO_2_), then placed in a chamber containing the same high-sucrose, low-magnesium aCSF composition at 32 °C for 30 min. After equilibrating for 30 min, the slices were transferred to another holding chamber in room temperature aCSF (in mM: 126 NaCl, 3 KCl, 1.25 NaH_2_PO_4_, 1.3 MgCl, 2.5 CaCl_2_, 26 NaHCO_3_, 10 glucose) saturated with 95%O_2_/5%CO_2_. Once transferred to the recording chamber, slices were continuously perfused with aCSF maintained at 32 °C. Borosilicate-glass recording electrodes (1–2 Mohms; A-M Systems) were pulled with a Sutter micropipette puller (Model P-97, Sutter Instrument, Novato, CA), and inserted in the *stratum radiatum* of hippocampal field CA1 to record field excitatory post-synaptic potentials (fEPSPs). To elicit evoked responses, current pulses applied with stimulus intensity adjusted to evoke ~ 50% of maximal fEPSPs (50 pA to 100 pA; 100 µs duration) at 30 s intervals was delivered using a bipolar stainless-steel stimulating electrode that was placed in the Schaffer collateral/commissural fibers. Electrical stimulation was delivered by an ISO-Flex isolator controlled by a Master eight pulse generator (AMPI, Jerusalem, Israel), triggered and recorded with a Multiclamp 700B amplifier (Molecular Devices, San Jose, CA). The slopes of fEPSPs were measured by linear interpolation from 20 to 80% of maximum negative deflection. Raw values of fEPSPs at half-maximal stimulus intensity (SI) during 15 min baseline recording were averaged to compare baseline excitability across groups. Data were analyzed with SciWorks (DataWave Technologies, Parsippany, NJ). The high-frequency theta burst stimulus (TBS) paradigm for induction of LTP consisted of 2 theta burst trains separated by 3 min, 10 bursts each, 5 pulses per burst, a burst frequency of 100 Hz and inter-burst interval of 200 ms. cAMP-dependent chemical LTP was induced by bath application of forskolin (10 μM) and rolipram (10 μM) for 20 min.

For analysis of paired-pulse inhibition/facilitation of population spikes in CA1 neurons, the stimulating electrode was placed in the *stratum radiatum* to stimulate Schaffer collaterals, and the recording electrode in CA1 *stratum pyramidale*. Population spike (PS) magnitudes were measured as the amplitude of the negative spike, extrapolated by drawing a tangent between the peak of the EPSP and the peak of the PS, and then taking the vertical distance from the negative peak of the PS to the tangent line. Population spikes were evoked at a stimulus intensity that elicited 50% of maximal amplitude in the first pulse spike. A series of inter-pulse intervals (IPIs), 10–1000 ms in duration, was used to study synaptic properties of facilitation or depression. Four sweeps of population spikes were recorded for each IPI per slice, and the responses averaged. The ratio of the second evoked population spike amplitude (PS2) to the first (PS1) was used to determine depression or facilitation, with a ratio > 1 corresponding to facilitation, and a ratio < 1 indicating depression.

### Statistical analyses

Two-way ANOVA with Repeated Measures (RM) followed by Tukey’s correction for multiple comparisons (GraphPad Prism, San Diego, CA) was used to determine the effect and interaction between dose and training in behavior analysis. One-way ANOVA with RM followed by Tukey’s correction for multiple comparisons was used to determine the effect of dose on LTP magnitude between groups. One-way ANOVA without repeated measures followed by Tukey’s correction for multiple comparisons was used for comparison of neurogenesis quantification between groups. Two-way ANOVA with repeated measures and Tukey’s correction for multiple comparisons was used when the effects of sex and dose were tested. Statistical significance was preset at *P* < 0.05.

## Supplementary Information

Supplementary Information.
