# Supplementary material for: Life-long brain compensatory responses to galactic cosmic radiation exposure
Source: Sci Rep. 2021 Feb 22;11:4292. doi: 10.1038/s41598-021-83447-y (PMC7900210; doi:10.1038/s41598-021-83447-y)
Supplement: Supplementary file 1 — Supplementary Information. [file 41598_2021_83447_MOESM1_ESM.docx]

**Life-Long Brain Compensatory Responses to Galactic Cosmic Radiation Exposure**

Omid Miry^1^, Xiao-lei Zhang^1^, Linnea R. Vose^1^, Katisha R. Gopaul^1^, Galadu Subah^1^, Juliet A. Moncaster^2,6^, Mark W. Wojnarowicz^3,6^, Andrew M. Fisher^4,6^, Chad A. Tagge^4,6^, Lee E. Goldstein^2,5,6^, Patric K. Stanton^1*^

**SUPPLEMENTARY INFORMATION**

**Supplementary Figure 1. Exposure to ^56^Fe particle radiation does not alter visual acuity acutely or chronically.** Visual acuity, measured by the threshold of spatial frequency between virtual grates which no longer elicits an opto-fixation reflex, is not affected by any dose of radiation exposure in either males at two **(a)** or 12 months **(b)**, or in females at two **(c)** or 12 months **(d)** post-exposure. *P* > 0.05 RM ANOVA, n=8 mice per group. Each bar represents mean spatial frequency ± SEM.

**Supplementary Figure 2. Exposure to ^56^Fe particle radiation does not elicit anxiety-related behavior acutely or chronically.** The average percent of time spent in the dark, closed arms of an Elevated Plus Maze does not differ significantly between doses in males at two months **(a)** or 12 months **(b)** post-exposure, nor does it differ in females two months (c) or 12 months **(d)** post-exposure. Each bar represents mean ± SEM. *P* > 0.05, one-way ANOVA, n=5 mice per group.

**Supplementary Figure 3. Exposure to ^56^Fe particle radiation does not alter locomotor function acutely or chronically.** The average distance traveled in an open field does not differ significantly between doses in males at two months **(a)** or 12 months **(c)** post-exposure. Each bar represents mean ± SEM. *P* > 0.05, one-way ANOVA, n=5 mice per group. The ratio of distance traveled in the center of the open field to the distance traveled in the periphery of the open field is an additional measure of anxiety and does not differ between doses at two months (b) or at 12 months post exposure (b). Each bar represents mean ± SEM. *P* > 0.05, one-way ANOVA, n=5 mice per group.
